# Reproductive Toxicity of a Mixture of Regulated Drinking-Water Disinfection By-Products in a Multigenerational Rat Bioassay

**DOI:** 10.1289/ehp.1408579

**Published:** 2015-02-19

**Authors:** Michael G. Narotsky, Gary R. Klinefelter, Jerome M. Goldman, Anthony B. DeAngelo, Deborah S. Best, Anthony McDonald, Lillian F. Strader, Ashley S. Murr, Juan D. Suarez, Michael H. George, E. Sidney Hunter, Jane Ellen Simmons

**Affiliations:** National Health and Environmental Effects Research Laboratory, Office of Research and Development, U.S. Environmental Protection Agency, Research Triangle Park, North Carolina, USA

## Abstract

**Background:**

Trihalomethanes (THMs) and haloacetic acids (HAAs) are regulated disinfection by-products (DBPs); their joint reproductive toxicity in drinking water is unknown.

**Objective:**

We aimed to evaluate a drinking water mixture of the four regulated THMs and five regulated HAAs in a multigenerational reproductive toxicity bioassay.

**Methods:**

Sprague-Dawley rats were exposed (parental, F_1_, and F_2_ generations) from gestation day 0 of the parental generation to postnatal day (PND) 6 of the F_2_ generation to a realistically proportioned mixture of THMs and HAAs at 0, 500×, 1,000×, or 2,000× of the U.S. Environmental Protection Agency’s maximum contaminant levels (MCLs).

**Results:**

Maternal water consumption was reduced at ≥ 1,000×; body weights were reduced at 2,000×. Prenatal and postnatal survival were unaffected. F_1_ pup weights were unaffected at birth but reduced at 2,000× on PND6 and at ≥ 1,000× on PND21. Postweaning F_1_ body weights were reduced at 2,000×, and water consumption was reduced at ≥ 500×. Males at 2,000× had a small but significantly increased incidence of retained nipples and compromised sperm motility. Onset of puberty was delayed at 1,000× and 2,000×. F_1_ estrous cycles and fertility were unaffected, and F_2_ litters showed no effects on pup weight or survival. Histologically, P_0_ (parental) dams had nephropathy and adrenal cortical pathology at 2,000×.

**Conclusions:**

A mixture of regulated DBPs at up to 2,000× the MCLs had no adverse effects on fertility, pregnancy maintenance, prenatal survival, postnatal survival, or birth weights. Delayed puberty at ≥ 1,000× may have been secondary to reduced water consumption. Male nipple retention and compromised sperm motility at 2,000× may have been secondary to reduced body weights.

**Citation:**

Narotsky MG, Klinefelter GR, Goldman JM, DeAngelo AB, Best DS, McDonald A, Strader LF, Murr AS, Suarez JD, George MH, Hunter ES III, Simmons JE. 2015. Reproductive toxicity of a mixture of regulated drinking-water disinfection by-products in a multigenerational rat bioassay. Environ Health Perspect 123:564–570; http://dx.doi.org/10.1289/ehp.1408579

## Introduction

Disinfection of drinking water is important in controlling microbial contamination in tap water and has proven to be a major advancement in public health. However, oxidizing disinfectants react with organic materials in the water, forming complex mixtures of disinfection by-products (DBPs). Although > 600 unique DBPs (halogenated and nonhalogenated) have been identified, approximately 50% of the mass of the halogenated DBPs remain unidentified. Epidemiological and animal toxicity studies have suggested that chlorination by-products may increase risk of adverse reproductive and developmental outcomes such as low birth weight, birth defects, spontaneous abortion, and stillbirth (Grazuleviciene et al. 2013; Levallois et al. 2012; Nieuwenhuijsen et al. 2009). However, inconsistent findings among epidemiological studies have yielded uncertainty regarding these associations (Hrudey 2009).

The most prevalent DBPs in chlorinated water are trihalomethanes (THMs) and haloacetic acids (HAAs) (Krasner et al. 2006; Weinberg et al. 2002). Four of the THMs (chloroform, bromodichloromethane, chlorodibromomethane, and bromoform) are regulated by the U.S. Environmental Protection Agency (EPA) in drinking water as a group (i.e., the concentrations of the individual DBPs are summed) at 80 μg/L, whereas five of the HAAs (chloroacetic, dichloroacetic, trichloroacetic, bromoacetic, and dibromoacetic) are regulated as a group at 60 μg/L (U.S. EPA 2006). These maximum contaminant levels (MCLs) are based on cancer data. Both classes of chemicals are regulated as a rolling annual average, that is, average levels over the previous four quarters are calculated each quarter.

Although THMs and HAAs have been evaluated individually for adverse reproductive and developmental effects, there are very limited examples of reproductive toxicity assessment of any mixtures involving these regulated DBPs (Narotsky et al. 2011). To our knowledge, these DBPs have never been evaluated for reproductive toxicity in the way they are regulated—as defined mixtures of the four regulated THMs and the five regulated HAAs in drinking water. Thus, using chemical proportions seen following water treatment by chlorination, we sought to assess the combined reproductive toxicity of the nine regulated chlorination DBPs in a multigenerational study in rats.

## Materials and Methods

*Chemicals*. Chloroform (lot no. 094K3725, purity ≥ 99%) was obtained from Sigma, bromodichloromethane (batch no. 30832, purity ≥ 98%) was obtained from Supelco, and dibromoacetic acid (lot no. 1126501, purity 99.1%) was obtained from Fluka. Chloroacetic acid (lot no. 05715PC, purity 99%), chlorodibromomethane (lot no. 03113BC, purity 98%), and bromoform (lot no. 13411HC, purity ≥ 99%) were obtained from Aldrich. Dichloroacetic acid (lot no. 13421HC, purity ≥ 99%), trichloroacetic acid (lot no. 015K0601, purity 99.9%), and bromoacetic acid (lot no. 07216LC, purity ≥ 99%) were obtained from Sigma-Aldrich. The vehicle was 0.25% Alkamuls^®^ EL-620 (lot no. SP1F090390; Rhodia Inc.) in reverse-osmosis purified deionized water.

Concentrations of each component DBP are listed in Table 1. Chemical proportions were based on those reported at the water utility that provided water for our whole-mixture toxicity studies (Narotsky et al. 2012, 2013); these proportions were held constant across dose levels (0, 500×, 1,000×, or 2,000× of the MCLs). Overall dose levels were selected based on results of a preliminary dose-range–finding study with pregnant and lactating rats.

**Table 1:** Concentrations of regulated trihalomethanes (THMs) and haloacetic acids (HAAs) for each treatment group.

Chemical	MCL (mg/L)	500× (mg/L)	1,000× (mg/L)	2,000× (mg/L)
Chloroform		22.39	44.77	89.54
Bromodichloromethane		12.98	25.96	51.92
Chlorodibromomethane		4.29	8.59	17.18
Bromoform		0.34	0.68	1.36
Chloroacetic acid		7.04	14.07	28.15
Dichloroacetic acid		13.52	27.03	54.06
Trichloroacetic acid		6.85	13.71	27.42
Bromoacetic acid		0.82	1.64	3.28
Dibromoacetic acid		1.77	3.54	7.09
Total THMs	0.08	40	80	160
Total HAAs	0.06	30	60	120
Total DBPs		70	140	280
Chemical proportions, based on those reported at the water utility that provided water for whole-mixture toxicity studies (Narotsky et al. 2012, 2013), were held constant across dose levels.				

Mixtures were prepared twice weekly. Prior to addition of THMs to the dosing solutions, pH was adjusted to values of 6–7 using sodium hydroxide. Dosing formulations not immediately placed on cages were stored at 4°C in light-protected polyethylene carboys.

*Animals and husbandry*. Timed-pregnant Sprague-Dawley rats (Charles River Laboratories, Raleigh, NC) were obtained on gestation day (GD) 0. GD 0 was defined as the day that evidence of mating (copulatory plug or vaginal sperm) was detected. The dams, weighing 165–245 g and 10–14 weeks of age, were housed individually in polycarbonate cages. At weaning, male progeny were housed two per cage and females were housed three per cage. Dams and weanlings were uniquely identified with eartags and provided heat-treated pine shavings for bedding. Animal rooms were maintained on a 12/12-hr light/dark cycle (lights on at 0600 hours). Room temperature and relative humidity were maintained at 22.2 ± 1.1°C and 50 ± 10%, respectively. Feed (Formulab Diet 5008; PMI^®^ LabDiet^®^) and drinking water were provided *ad libitum*. Water was provided in amber glass bottles with Teflon®-lined caps and stainless steel sipper tubes equipped with stainless steel ball bearings. Animals used in this study were treated humanely and with regard for the alleviation of suffering. Procedures were approved by the Institutional Animal Care and Use Committee, and animals were maintained in a facility certified by the American Association for the Accreditation of Laboratory Animal Care.

*Experimental design*. Twenty-five parental (P_0_) animals were assigned to each treatment group using a nonbiased randomization procedure that assured a homogeneous distribution of body weight (Narotsky et al. 1997). The designated DBP mixture was the sole source of drinking water for the animals in each treatment group. Control animals received vehicle. The dams were exposed to their designated water through the weaning of their litters. The progeny (F_1_ generation) continued their exposure past puberty and breeding, through gestation of the F_1_ females, and up to postnatal day (PND) 6 of the F_2_ litters (see Supplemental Material, Figure S1).

*Procedures*. Body weights and water consumption. Body weights were recorded at least twice per week throughout the experiment. Water consumption for each cage was also recorded at least twice per week.

Parturition. Beginning on GD20, dams were observed periodically to determine the time of parturition. The stage of parturition (completed, in progress, first pup delivered) was also recorded.

Litter examinations. F_1_ litters were examined on PNDs 0 (day of birth), 6, 13, 21, and 26. F_2_ litters were examined on PNDs 0 and 6. On PND0, pups were examined for evidence of nursing (i.e., abdominal milk bands) and were sexed, counted, and weighed. In addition, 15 F_1_ litters each from the control group and the high-dose group (2,000×) were selected randomly, and the anogenital distance (AGD) of each pup was measured as described previously (Narotsky et al. 2013). On PND6, pups were again sexed, counted, and weighed. F_1_ litter sizes were reduced on PND6 to a maximum of 10 pups (5 males and 5 females when possible). On PND13, each F_1_ pup was examined for eye opening and nipple retention, as described previously (Narotsky et al. 2013). On PND21, F_1_ pups were sexed and weighed. Because of low pup weights in the high-dose group, weaning of all pups was delayed until PND26; at this time pups were sexed, weighed, and weaned.

Selection of weanlings. F_1_ weanlings were randomly selected for different roles as the experiment continued (see Supplemental Material, Table S1). From each litter, one male and one female (designated as “A” animals) were selected for breeding to produce F_2_ litters. An additional male and female (“B” animals) were selected for examination of serum hormones at puberty; the males were killed on PND55, whereas the females were killed on the day of vaginal opening (VO). A third female (“C”) was killed on the day of estrus for examination of serum hormones. For 10 randomly selected litters per group, one male (“C”) was selected to provide epididymal sperm for *in utero* insemination, and another male (“D”) was bred to two untreated females.

Pubertal examinations. F_1_ animals were examined daily for onset of puberty. Females were examined for VO starting on PND27 and were scored as closed, partially open, or fully open. On the day of VO (i.e., when fully open), each female was weighed; B-females were killed by decapitation and sera were collected for assay of progesterone, estradiol, and leptin.

Males were examined for preputial separation (PPS) starting on PND34. Males were weighed on the day of PPS and daily on PNDs 41–47. PPS was scored as none, minimal, at least 50%, or complete. On PND55, B-males were weighed and killed, sera were collected for measurement of testosterone, and testes and epididymides were weighed and fixed (Narotsky et al. 2013).

Estrous cycles. For 19 days beginning on PND46 or PND47, daily vaginal smears of A-females were examined microscopically for vaginal cytology. Estrous cycles were classified as regular (4 or 5 days), extended, or abnormal (Goldman et al. 2007). During the third week of this period, PNDs 57–65, C-females were killed on the day of estrus and evaluated for serum levels of estradiol and progesterone. Selected C-females, 10–11/group, were also evaluated for the number of released oocytes; oviducts were excised and oocytes were flushed and counted as previously described (Perreault and Mattson 1993).

Breeding. Following estrous cycle monitoring, each A-female was transferred to the cage of a randomly selected nonsibling A-male of the same treatment group for up to 14 days. When evidence of mating (copulatory plug or vaginal sperm) was observed (GD0), the female was weighed and singly housed.

Similarly, D-males were cohabited with two untreated females for up to 7 days. These females were necropsied on GDs 9–14 to evaluate pregnancy status. Uteri were examined for the numbers of live and resorbed implantation sites, and ovaries were examined for the number of corpora lutea.

*In utero* insemination. C-males, 10/group, were bred to untreated receptive females using artificial insemination as previously described (Narotsky et al. 2013). Briefly, within 15 min of sperm diffusion from the proximal cauda epididymis, each uterine horn of the anesthetized recipient female was injected with a volume containing 5 × 10^6^ sperm, a value that results in approximately 75% fertility of control males. A single female was inseminated per male. Nine days later, inseminated females were killed, and corpora lutea (reflecting the number of ovulations) and uterine implantation sites were counted. The fertility of each male was expressed as implants divided by corpora lutea.

Sperm measures. Cauda epididymal sperm motility and morphology were evaluated as described previously (Klinefelter et al. 2002) in adult (PNDs 89–93) males. In males assessed for fertility by artificial insemination (PNDs 96–100), SP22, a sperm membrane protein and biomarker of fertility (Klinefelter 2008), was quantified using an enzyme-linked immunosorbent assay (ELISA).

Necropsies. Full necropsies were conducted on P_0_ females at 26 days postpartum (upon weaning of their litters), on F_1_ A-males at PNDs 89–93, and on F_1_ A-females at PNDs 96–104 (after PND6 examinations of the F_2_ litters). Animals were weighed and killed by decapitation; trunk blood was collected and sera were prepared. Sera were frozen at –80°C for hormone analysis. Cranial, thoracic, abdominal, and pelvic viscera were examined grossly. Organ weights were recorded for the brain, kidneys, spleen, ovaries, testes, thymus, liver, lung, adrenal glands, pituitary gland, uterus with oviducts and cervix, epididymides, prostate, and seminal vesicles with coagulating glands (and fluids). Uterine implantation sites were counted. Uteri from nonparous females were stained with 2% ammonium sulfide to detect cases of full-litter resorption (Narotsky et al. 1997). Tissues from liver, lungs, kidneys, adrenals, thymus, spleen, stomach, duodenum, ileum, cecum, colon (proximal, middle, distal), mesenteric lymph nodes, trachea, esophagus, thyroid, pituitary gland, urinary bladder, prostate, seminal vesicle and coagulating gland, vagina, and ovaries, were fixed in buffered formalin.

For males, the left testis and epididymis were fixed in Bouin’s fluid, and the right cauda epididymis was sampled for assessment of sperm motility and morphology.

Histology. For P_0_ females, F_1_ A-males, and A-females, fixed tissues from 10 randomly selected rats from the control and high-dose groups were embedded in paraffin blocks, sectioned, stained with hematoxylin and eosin, and examined microscopically. If results from this initial examination suggested a treatment effect, the specified tissue of the remaining rats were also processed and examined.

For 10–11 F_1_ males and females per group, three colon segments were stained with new methylene blue for analysis of aberrant crypt foci (DeAngelo et al. 2002).

For P_0_ and F_1_ A-females, the ovaries were examined quantitatively for primordial and primary follicles by examining 20 cross sections (5 μm thick) per ovary. Routine histopathological examination of the ovaries was conducted in conjunction with the enumeration of follicles.

*Hormone measurements*. Estradiol, progesterone, and testosterone were analyzed by antibody-coated tube radioimmunoassay using Coat-a-Count^®^ kits (Diagnostic Products). Intra-assay coefficients of variation for quality control samples were 9.1, 5.4, 1.5, and 2.1 for estradiol, progesterone, leptin, and testosterone, respectively. All testosterone samples were quantified in a single assay. For estradiol, progesterone, and leptin, the interassay coefficients of variation were 7.2, 1.7, and 2.6, respectively.

*Statistics*. Inferential statistical tests used a significance level of 0.05; adjustments were not made for multiple end points.

For all developmental and reproductive data, the litter was considered the experimental unit; for example, litter means and frequencies per litter were used as the experimental units for analyzing pup weights and pup examination data. Prenatal loss (the number of implants minus the number of viable pups at PND0), neonatal loss (the number of pups viable on PND0 but not on PND6), and perinatal loss (the number of implants minus the number of live pups on PND6) were analyzed as percentages of the number of implants (prenatal and perinatal loss) or the number of live pups at PND0 (neonatal loss).

Continuous data, counts per litter, and proportions per litter were evaluated by analysis of variance (ANOVA) using the general linear models (GLM) procedure in SAS, Release 9.1 (SAS Institute Inc.). Proportions per litter (e.g., prenatal loss) underwent arcsine square root transformation prior to GLM analysis. End points pertaining to survival were analyzed using one-tailed tests. Gestation lengths were analyzed using the Kruskall–Wallis test. Pup weight analysis used the number of live PND0 pups as a covariate. Similarly, analyses of the numbers of live pups used the number of implants as a covariate. Incidences per group (e.g., estrous cycles) were analyzed with Fisher’s exact test to compare each group with controls.

AGD was analyzed by analysis of covariance with pup weight as a covariate and litter as a random effect using Proc Mixed (SAS).

Because of the potential bias inherent in the use of birth-based age for assessment of onset of puberty (Narotsky 2011), pubertal data were analyzed using both conception-based age and day-of-birth–based age. Birth-based age was defined as the number of days since birth that PPS or VO were observed, whereas conception-based age was defined as the number of days since GD22 that these landmarks were observed, regardless of the actual day of parturition.

## Results

**Figure 1:**
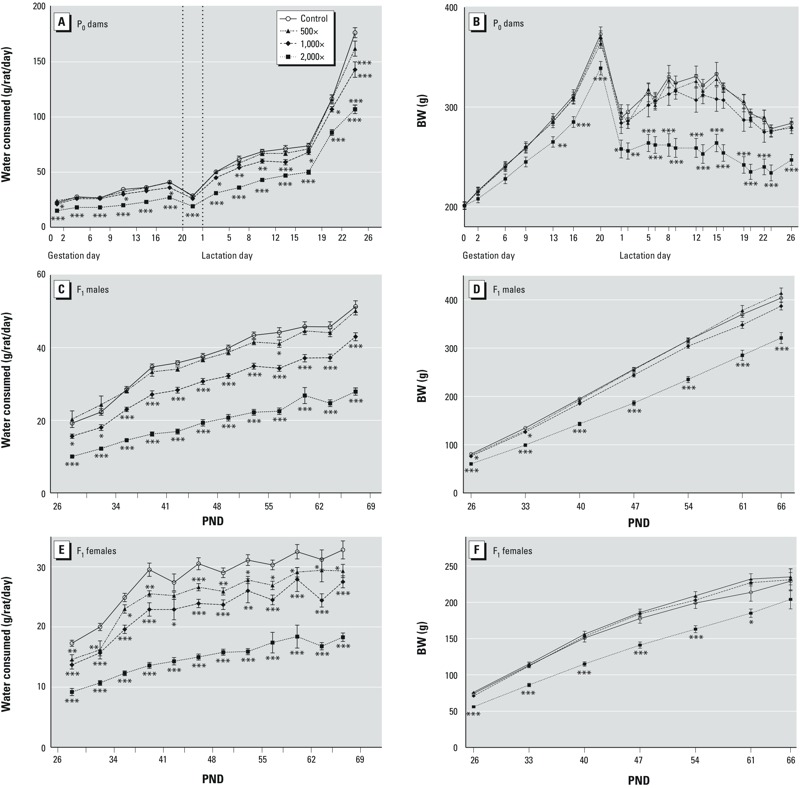
Mean (± SE) water consumption (*A*,*C*,*E*) and body weight (BW; *B*,*D*,*F*) for P_0_ females (*A,B*; 24–25/group), F_1_ males (*C,D*; 24–25/group), and F_1_ females (*E,F*; 24/group). For water consumption, data points represent consumption for an interval, which are demarcated by ticks on the horizontal axis; data are plotted at the interval’s midpoint. *p < 0.05, **p < 0.01, and ***p < 0.001 compared with controls.

*P_0_ dams*. Maternal body weights (Figure 1B) and water consumption (Figure 1A) of P_0_ females were significantly reduced compared with controls throughout gestation and lactation in the group receiving 2,000× water. At 1,000×, body weights were comparable with those of controls but water consumption was significantly reduced intermittently during gestation and consistently throughout lactation. At 500×, body weights and water consumption were comparable to those of controls. Pregnancy rates were ≥ 96% in all groups, and all dams successfully maintained their pregnancies to term (see Supplemental Material, Table S2). Gestation lengths were comparable for all groups; all dams delivered on GD21 or GD22, and no abnormalities in parturition were noted.

**Figure 2:**
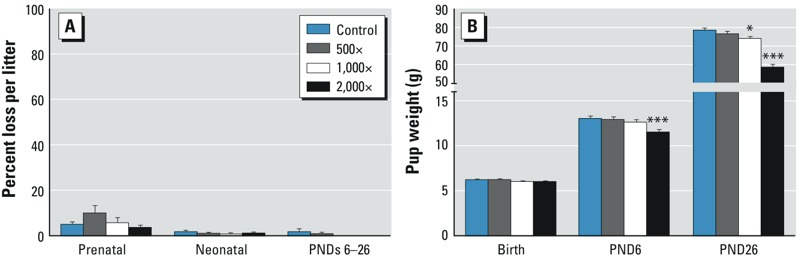
F_1_ pup mortality and pup weight. (*A*) Prenatal loss (implantation to birth), neonatal loss (birth to PND6), and postnatal loss from PNDs 6–26 showed no adverse effects of treatment. (*B*) F_1_ pup weights were unaffected at birth, but reduced pup weights were seen beginning on PND6. Bars represent mean ± SE per litter (24–25 litters/group). For complete numeric data, see Supplemental Material, Table S2. *p < 0.05, and ***p < 0.001, compared with controls.

*F_1_ litters*. No pup malformations were observed at any of the F_1_ litter examinations, and no treatment effects on viability were evident. The numbers of uterine implantation sites were comparable across all groups, as were the numbers of pups at each postnatal examination (see Supplemental Material, Table S2). Attrition rates (i.e., prenatal loss, postnatal loss) were unaffected by treatment (Figure 2A; see also Supplemental Material, Table S2). Although pup weights (Figure 2B) were comparable between groups at PND0, at all subsequent litter examinations pup weights were significantly reduced in the 2,000× group (Figure 2; see also Supplemental Material, Table S2). In the 1,000× group, pup weight reductions were significant in males at PND26 (weaning) and in females at PNDs 21 and 26 (see Supplemental Material, Table S2). Pup weights at 500× were unaffected at all litter examinations.

All pups in 15 litters from the control and high-dose groups were each examined for AGD at the PND-0 examination. Values were comparable between the groups for both male and female progeny (see Supplemental Material, Table S2).

On PND13, F_1_ pups were examined for eye opening and nipple retention (see Supplemental Material, Table S3). The incidences of pups with both eyes open, or both eyes closed, were comparable for all groups. Males that still had nipples were observed only in the treated groups. The mean ± SE percents affected per litter were 3.2 ± 1.9, 1.0 ± 1.0, and 6.0 ± 2.3 at 500×, 1,000×, and 2,000×, respectively. The incidence at 2,000× was significantly different from controls. For females, less-than-prominent nipples were observed only at 2,000×, but this incidence did not reach significance.

*F_1_ animals postweaning*. Body weights and water consumption (Figure 1) of F_1_ males and females postweaning were significantly reduced at 2,000×. At lower concentrations, female body weights (Figure 1D) were comparable to controls, whereas male body weights (Figure 1F) at 1,000× were significantly reduced only 1 week postweaning. Water consumption, however, was significantly reduced for both males (Figure 1C) and females (Figure 1E) at 1,000×. At 500×, female water consumption was reduced compared with controls at most intervals, but male consumption was significantly reduced only during the PND 55–58 interval.

**Figure 3:**
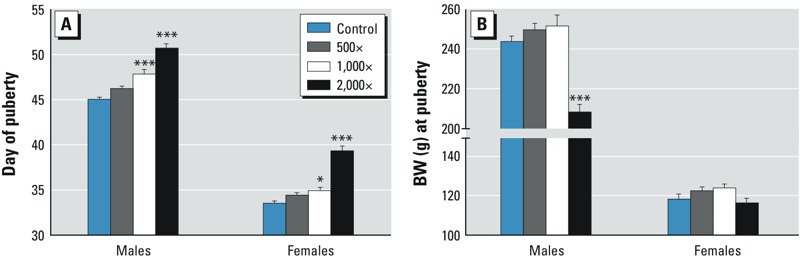
Pubertal data. (*A*) Vaginal opening, a marker for the onset of puberty, was significantly delayed in high-dose females. The age of preputial separation, a marker for onset of puberty in males, was significantly increased in the middle- and high-dose groups. (*B*) Body weight (BW) on the day of puberty was significantly reduced in high-dose males. Bars represent mean ± SE per litter (24–25 litters/group). For complete numeric data, see Supplemental Material, Table S4. Ages are expressed as days after GD22. *p < 0.05, and ***p < 0.001, compared with controls.

Onset of male puberty, indicated by the day of PPS, was significantly delayed at 1,000× and 2,000×, whereas a nonsignificant delay (*p* = 0.0588) was noted at 500× (Figure 3A; see also Supplemental Material, Table S4). Compared with controls, PPS was delayed 1.2, 2.8, and 5.7 days at 500×, 1,000×, and 2,000×, respectively. Body weights on the day of PPS were reduced only at 2,000× (Figure 3B; see also Supplemental Material, Table S4). Hormone measurements from males necropsied on PND55 (when most males have reached puberty) revealed comparable serum testosterone levels across groups; however, significantly reduced concentrations of testosterone (< 50% of control) were observed in the testicular interstitial fluid at 2,000× (see Supplemental Material, Table S4).

For the females, onset of puberty, as indicated by the day of VO, was significantly delayed at 1,000× and 2,000× (Figure 3A; see also Supplemental Material, Table S4). Compared with controls, VO delays were 0.9, 1.4, and 5.8 days at 500×, 1,000×, and 2,000×, respectively. Body weights on the day of VO were comparable across groups (Figure 3B; see also Supplemental Material, Table S4). Serum samples obtained on the day of VO revealed comparable levels of leptin and estradiol across groups, but progesterone levels were significantly reduced (~ 50% of control values) at 1,000× and 2,000× compared with controls (see Supplemental Material, Table S4). Leptin, an adipose hormone important in regulating food intake and metabolism and with a permissive role in the onset of puberty (Sanchez-Garrido and Tena-Sempere 2013), was significantly correlated with body weight at 1,000× (*R*^2^ = 0.236, *p* < 0.05) and 2,000× (*R*^2^ = 0.457, *p* < 0.001).

Examination of vaginal cytology of two F_1_ females per litter for 19 days revealed regular 4- or 5-day cycles for all females except for females from one, two, four, and five litters at 0, 500×, 1,000×, and 2,000×, respectively. Among animals with irregular cycles, extended/abnormal diestrus was observed in two control littermates, two females (non-littermates) at 500×, five females (four litters) at 1,000×, and three females (three litters) at 2,000×. Extended estrus was observed in two females (two litters) at 2,000×. All incidences were comparable across groups.

For those females exhibiting regular estrous cycles, serum concentrations of progesterone and estradiol on the day of estrus were comparable across groups, as were the numbers of oocytes obtained from flushed oviducts (data not shown).

*Breeding of F_1_ animals*. During the 14-day breeding period, F_1_ breeding pairs showed no effects of treatment. One pair at 1,000× failed to mate, whereas all remaining pairs mated, most within the first 4 days of cohabitation (see Supplemental Material, Table S5). Pregnancy rates were comparable in all groups; all females were pregnant except for two controls and two females at 1,000×. All F_1_ dams delivered on GD21 or GD22, and gestation lengths were comparable across groups. No abnormalities in parturition were noted for the F_1_ dams.

**Figure 4:**
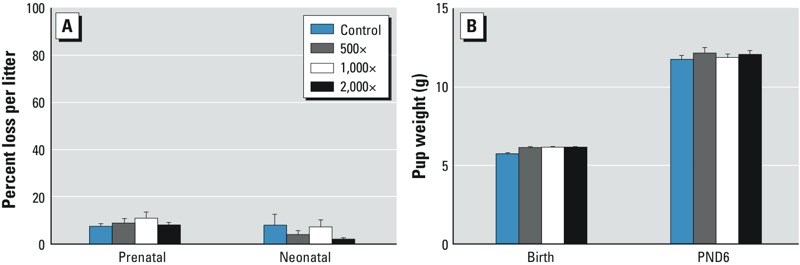
F_2_ pup mortality and pup weight. (*A*) Prenatal (implantation to birth) and neonatal (birth to PND6) loss showed no adverse effects of treatment. (*B*) F_2_ pup weights were unaffected at birth and PND6. Bars represent mean ± SE per litter (22–24 litters/group). For complete numeric data, see Supplemental Material, Table S5.

*F_2_ litters*. Examination of F_2_ litters on PNDs 0 and 6 showed no treatment effects. The numbers of implantation sites and live pups at each examination were comparable between controls and treated litters (see Supplemental Material, Table S5). Except for one control litter, all litters survived to PND6. Attrition rates per litter (prenatal loss, neonatal loss) (Figure 4A; see also Supplemental Material, Table S5) and pup weights (Figure 4B; see also Supplemental Material, Table S5) were comparable between groups. AGD, examined in the control and 2,000× groups on PND0, showed no differences between groups for either males or females. Except for filamentous tail observed in one control male, no malformations were observed in any of the F_2_ pups.

*Natural breeding of treated males to untreated females*. Ten F_1_ males from each group were each cohabited with two untreated females for up to 7 days. All males mated with at least one female. The incidences of males mating, impregnating females, and siring live litters were comparable in all groups (see Supplemental Material, Table S6). Midgestation examination of the females revealed comparable numbers of corpora lutea, implantation sites, live embryos, and resorption sites for all groups, as well as comparable attrition rates both pre- and postimplantation.

In utero *insemination*. Ten F_1_ males from each group provided cauda epididymal sperm samples to inseminate untreated females *in utero*. There were nonsignificant increases in preimplantation loss and the incidence of infertile males (see Supplemental Material, Table S7) with increasing dose. Sperm from these males showed nonsignificant (*p* ≤ 0.0567) reductions in SP22, a sperm protein biomarker of fertility, at 500× and 1,000×, whereas values at 2,000× were comparable to controls.

*Necropsies and histology*. Full necropsies were conducted on the P_0_ dams as well as on the F_1_ males and females that were used for breeding. No gross necropsy findings were attributed to treatment. For F_1_ males, absolute—but not relative—organ weights for brain, pituitary, liver, kidneys, adrenals, thymus, and spleen were reduced at 2,000×, and for thymus at 500× (see Supplemental Material, Table S8). For F_1_ females, absolute adrenal and liver weights were reduced in the high-dose group and relative kidney weights were increased at 1,000× and 2,000×.

Histological examinations of tissues from 10 randomly selected P_0_ rats from the control and 2,000× groups prompted follow-up examinations of adrenals and kidneys of all P_0_ females. Incidences of nephropathy and, in the adrenal cortex, hypertrophy of the zona glomerulosa and atrophy of the zona reticularis were significantly increased at 2,000× compared with controls (see Supplemental Material, Table S9), with severity increasing with dose for the adrenal observations. For F_1_ animals, histological examinations of 10 randomly selected males and females of the control and 2,000× groups revealed no findings attributed to treatment.

For 10–11 F_1_ animals per sex per group, colon samples (proximal, medial, and distal) were examined histologically for aberrant crypt foci; none were observed.

Histological examination of ovaries of 10 females each in the control and 2,000× groups of P_0_ and F_1_ dams revealed comparable numbers of primordial and primary follicles across groups (data not shown).

**Figure 5:**
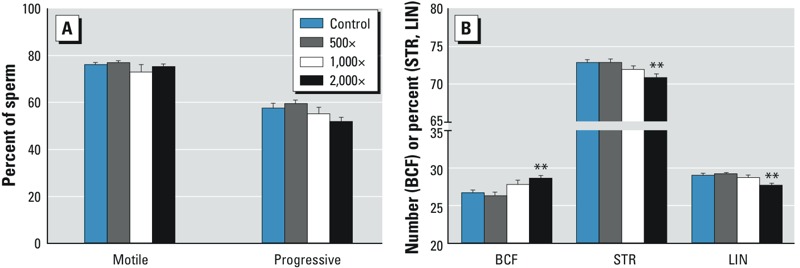
Cauda epididymal sperm motion parameters. (*A*) Percentages of motile and progressively motile sperm were unaffected. (*B*) In the 2,000× group, beat cross frequency (BCF) was significantly increased, and straightness (STR) and linearity (LIN) were significantly decreased. STR = straight line velocity/average path velocity × 100; LIN = straight line velocity/curvilinear velocity × 100; BCF = number of oscillations across mean trajectory. Values represent mean ± SE (23–25 males/group). **p < 0.01, compared with controls.

*Sperm motility*. Evaluations of sperm motion in adult F_1_ males indicated no effect on the percentage of motile sperm (Figure 5A); however, compromised forward motion at 2,000× was observed, including a significant increase in beat cross frequency (rate of crossing the average path trajectory) and decreases in straightness (linearity of the spatial average path) and linearity (linearity of the curvilinear trajectory) (Figure 5B).

## Discussion

Exposure to a mixture of regulated chlorination DBPs at 500× the MCLs had no adverse effects on any reproductive end points examined. Furthermore, exposure at up to 2,000× did not affect pregnancy maintenance, gestation length, prenatal survival, postnatal survival, or birth weights. In view of epidemiological associations reported for low birth weight and spontaneous abortion in humans exposed to chlorinated water (Levallois et al. 2012; Niewenhuijsen et al. 2009), the lack of effects of this subset of DBPs on birth weight and prenatal loss is noteworthy.

Although birth weights were unaffected, reduced maternal water consumption during lactation may have contributed to reduced pup weights as lactation progressed. In the 2,000× group, reduced weights were evident at PND6 and persisted to adulthood, whereas in the 1,000× group, reduced weights reached significance only between PND21 and PND33. Poor palatability of the DBP mixture at the higher dose levels may have contributed to reduced maternal water consumption, and in turn to the renal and adrenal pathology noted in the P_0_ dams and stunted growth in the progeny. Furthermore, it is possible that reproductive findings at the higher dose levels may also be secondary to decreased water consumption and body weights. Although AGD was unaffected, endocrine-related effects in F_1_ animals included a subtle increase in retained nipples in males at 2,000×, delayed onset of puberty in both males and females at 1,000× and 2,000×, and altered sperm motility parameters at 2,000×. F_1_ estrous cycles, breeding, fertility, and SP22 (a sperm protein biomarker of fertility) were unaffected, and F_2_ litters showed no effects on pup weight or viability.

In toxicity tests with individual chemicals, two regulated DBPs (dibromoacetic acid at 400 mg/L, bromodichloromethane at 150 mg/L) have been shown to delay puberty in rats (Christian et al. 2002a, 2002b; Klinefelter et al. 2004) and may have contributed to the effects seen here. Dibromoacetic acid has also been shown to reduce sperm quality in rats at 40 mg/L (Klinefelter et al. 2004) or 2 mg/kg by gavage (Kaydos et al. 2004; Klinefelter et al. 2004) and rabbits at 1 mg/kg in drinking water (Veeramachaneni et al. 2007). The lack of an effect on fertility in the present study was unsurprising because sperm quality in rats, unlike humans, must be substantially reduced to affect fertility (Klinefelter et al. 1994). However, *in utero* insemination with artificially reduced sperm numbers also showed no clear effect on fertility. *In vitro*, dibromoacetic acid has been shown to decrease progesterone secretion in newly matured ovarian follicles (Goldman and Murr 2002) and may have contributed to the reduced pubertal progesterone levels observed here.

In conjunction with the present study, we have also conducted a similar multigenerational study examining the effects of an environmental “whole” mixture concentrate of DBPs (i.e., containing unidentified and identified DBPs) (Narotsky et al. 2013). Consistent with the current study, the whole mixture significantly delayed puberty in females and had reduced sperm counts in males. Together, these studies provide valuable insight toward understanding the potential health risks of DBPs in tap water.

## Conclusions

A mixture of the regulated THMs and HAAs at concentrations 500× greater than regulatory MCLs had no adverse effects; furthermore, 2,000×, the highest concentration evaluated, did not affect the animals’ ability to reproduce. The lack of effects on prenatal survival and birth weight in this study contrast with associations reported in some epidemiological studies (Levallois et al. 2012; Nieuwenhuijsen et al. 2009). Although reproduction per se was unaffected, retained nipples and sperm motility effects in males at 2,000× and pubertal delays in both sexes at ≥ 1,000× the regulatory MCLs indicate that a mixture of these regulated DBPs can influence endocrine physiology; however, these findings may have been secondary to reduced water consumption and body weight.

## Supplemental Material

(405 KB) PDF
