# Supplementary material for: Reproductive Toxicity of a Mixture of Regulated Drinking-Water Disinfection By-Products in a Multigenerational Rat Bioassay
Source: Environ Health Perspect. 2015 Feb 19;123(6):564–70. doi: 10.1289/ehp.1408579 (PMC4455591; doi:10.1289/ehp.1408579)
Supplement: (405 KB) PDF [file ehp.1408579.s001.508.pdf]

Environ Health Perspect
doi: 10.1289/ehp.1408579

Supplemental Material

Reproductive Toxicity of a Mixture of Regulated Drinking-Water Disinfection By-Products in a Multigenerational Rat Bioassay

Michael G. Narotsky, Gary R. Klinefelter, Jerome M. Goldman, Anthony B. DeAngelo, Deborah S. Best, Anthony McDonald, Lillian F. Strader, Ashley S. Murr, Juan D. Suarez, Michael H. George, E. Sidney Hunter III, and Jane Ellen Simmons

Table S1. Disposition of F_1 weanlings on PND 26. From each litter, one A-male and one A-female were selected to breed, one B-male and one B-female were killed at puberty (males on PND 55, females on the day of vaginal opening), and one C-female was killed on the day of estrus. From 10 litters per group, one C-male was used for artificial insemination of untreated females and one D-male was bred naturally to two untreated females.

Fate Designation	Litters per group	Males	Females
A	25	Breed (produce F_2)	Breed (produce F_2)
B	25	Pubertal necropsy, PND 55	Pubertal necropsy
C	10	Artificial insemination	Estrus necropsy
D	10	Breed to untreated females	

Table S2. Summary of reproductive and developmental data for P_0 dams and their F_1 litters.

Variable	Control	500×	1000×	2000×
**Number of females**				
Received	25	25	25	25
Pregnant	24	25	24	24
Delivered GD 21	12	14	15	11
Delivered GD 22	12	11	9	13
With live litter PND 0	24	25	24	24
With live litter PND 26	24	25	24	24
**Mean ± S.E. number per litter**				
Implantation sites	13.2 ± 0.6	13.6 ± 0.4	14.0 ± 0.4	13.3 ± 0.6
Live pups				
PND 0	12.9 ± 0.6	12.4 ± 0.6	13.4 ± 0.4	13.5 ± 0.3
PND 6	12.7 ± 0.5	12.3 ± 0.6	13.3 ± 0.4	13.3 ± 0.4
PND 21[a]	9.5 ± 0.2	9.4 ± 0.3	9.9 ± 0.1	10.0 ± 0.0
PND 26	9.5 ± 0.2	9.4 ± 0.3	9.9 ± 0.1	10.0 ± 0.0
**Mean ± S.E. percent per litter**				
Males at PND 0	51.4 ± 2.7	50.3 ± 3.2	49.3 ± 2.7	48.1 ± 2.5
Prenatal loss	4.9 ± 1.1	9.9 ± 3.4	5.5 ± 2.4	3.5 ± 1.0
Postnatal loss, PND 0-6	1.6 ± 0.7	0.9 ± 0.5	0.6 ± 0.6	1.0 ± 0.6
Postnatal loss, PND 6-21	1.7 ± 1.3	0.8 ± 0.6	0	0
Postnatal loss, PND 21-26	0	0	0	0
**Mean ± S.E. pup weight (g) per litter**				
Males				
PND 0	6.4 ± 0.1	6.3 ± 0.1	6.1 ± 0.1	6.2 ± 0.1
PND 6	13.3 ± 0.3	13.2 ± 0.3	12.9 ± 0.3	11.7 ± 0.3**
PND 21	53.7 ± 1.3	53.1 ± 1.3	50.7 ± 0.8	40.5 ± 1.3***
PND 26	81.2 ± 1.3	79.6 ± 1.6	76.3 ± 1.1*	59.9 ± 1.6***
Females				
PND 0	6.0 ± 0.1	5.9 ± 0.1	5.8 ± 0.1	5.8 ± 0.1
PND 6	12.8 ± 0.3	12.6 ± 0.3	12.3 ± 0.3	11.3 ± 0.3**
PND 21	52.1 ± 1.1	51.3 ± 1.0	48.9 ± 0.9*	39.5 ± 1.3***
PND 26	75.9 ± 1.2	74.4 ± 1.3	71.5 ± 1.1*	57.3 ± 1.5***
**Mean ± S.E. anogenital distance (mm)[b]**				
Males	3.32 ± 0.06	--	--	3.34 ± 0.05
Females	1.43 ± 0.03	--	--	1.38 ± 0.02

*Significantly different from control value ($p<0.05$).

**Significantly different from control value ($p<0.01$).

***Significantly different from control value ($p<0.001$).

[a]Litters were reduced to 10 pups after the PND-6 examination. [b]All pups in 15 randomly selected litters from the control and high-dose groups were evaluated for anogenital distance on PND 0. Values represent the mean ± S.E. per litter.

Table S3. Summary of F_1 Day-13 litter examinations of eye opening and nipple retention.

**Variable**	**Control**	**500×**	**1000×**	**2000×**
Number of litters	24	25	24	24
**Mean ± S.E. per litter**				
Both eyes fully closed (%)	64.2 ± 8.1	67.8 ± 7.6	69.6 ± 7.1	74.2 ± 6.8
Both eyes fully open (%)	0.4 ± 0.4	0.4 ± 0.4	0	0
Males				
Number of nipples	0	0	0	0.1 ± 0.1
Males with nipples (% affected)	0	3.2 ± 1.9	1.0 ± 1.0	6.0 ± 2.3*
Females (% affected)				
with less-than-prominent nipple	0	0	0	5.8 ± 4.3
with supernumerary nipple	0	0	0	0

*Significantly different from control value ($p<0.05$).

Table S4. Summary of pubertal data for F_1 progeny.

**Variable**	**Control**	**500×**	**1000×**	**2000×**
Number of litters	24	25	24	24
**Mean ± S.E. per litter**				
Males				
Age at Puberty (preputial separation)				
Days since GD 22	45.0 ± 0.3	46.2 ± 0.3	47.8 ± 0.6***	50.7 ± 0.5***
Days since day of birth	45.5 ± 0.3	46.8 ± 0.3	48.4 ± 0.6***	51.2 ± 0.5***
Body weight (g) on day of puberty	243.5 ± 2.9	249.4 ± 3.4	251.4 ± 5.6	208.1 ± 4.3***
Serum Testosterone (ng/ml)	2.3 ± 0.3	2.3 ± 0.3	2.1 ± 0.2	1.6 ± 0.2
Interstitial Fluid Testosterone (ng/ml)	118.6 ± 21.5	120.4 ± 16.9	126.1 ± 17.7	51.4 ± 7.3**
Females				
Age at Puberty (vaginal opening)				
Days since GD 22	33.5 ± 0.3	34.4 ± 0.3	34.9 ± 0.4*	39.3 ± 0.6***
Days since day of birth	34.0 ± 0.4	34.9 ± 0.3	35.5 ± 0.4*	39.8 ± 0.7***
Body weight (g) on day of puberty	118.0 ± 2.7	122.3 ± 2.1	123.8 ± 2.2	116.1 ± 2.6
Serum Estradiol (pg/ml)	23.3 ± 2.0	24.2 ± 1.5	23.8 ± 2.0	23.1 ± 2.1
Serum Progesterone (ng/ml)	11.2 ± 2.2	8.0 ± 1.2	5.6 ± 1.0*	6.1 ± 0.9*
Serum Leptin (ng/ml)	1.8 ± 0.1	1.7 ± 0.1	1.6 ± 0.1	1.5 ± 0.1

*Significantly different from control value ($p<0.05$).

**Significantly different from control value ($p<0.01$).

***Significantly different from control value ($p<0.001$).

Note: Age at puberty is presented as a conception-based (days since GD 22) as well as a birth-based value.

Table S5. Summary of reproductive and developmental data for F_1 breeding pairs and their F_2 litters.

Variable	Control	500×	1000×	2000×
**Number of F_1 females**				
Bred	24	24	24	24
Cohabitation days to mate: 1-4	21	22	21	23
Cohabitation days to mate: 5-10	3	2	1	1
Cohabitation days to mate: 11-14	0	0	1	0
Did not mate	0	0	1	0
Pregnant	22	24	22	24
Delivered GD 21	17	14	15	14
Delivered GD 22	5	10	7	10
With live litters PND 0	22	24	22	24
With live litters PND 6	21	24	22	24
**Mean ± S.E. number per litter**				
Implantation sites	16.4 ± 0.4	15.8 ± 0.4	16.5 ± 0.4	14.2 ± 0.5
Live pups PND 0	15.2 ± 0.4	14.5 ± 0.5	14.8 ± 0.6	13.1 ± 0.5
Live pups PND 6	14.0 ± 0.9	13.8 ± 0.5	13.7 ± 0.7	12.8 ± 0.5
**Mean ± S.E. percent per litter**				
Percent males at PND 0	52.1 ± 3.2	52.3 ± 2.6	52.3 ± 2.8	51.9 ± 3.1
Prenatal loss	7.1 ± 1.4	8.5 ± 2.2	10.5 ± 3.0	7.6 ± 1.4
Neonatal loss, PND 0-6	7.6 ± 4.9	3.6 ± 1.9	6.8 ± 3.4	1.6 ± 1.0
**Mean ± S.E. pup weight (g) per litter**				
Males PND 0	5.9 ± 0.1	6.3 ± 0.1	6.2 ± 0.1	6.2 ± 0.1
Males PND 6	11.9 ± 0.3	12.4 ± 0.4	12.0 ± 0.3	12.2 ± 0.3
Females PND 0	5.6 ± 0.1	5.9 ± 0.1	6.0 ± 0.1	5.9 ± 0.1
Females PND 6	11.4 ± 0.3	11.7 ± 0.4	11.6 ± 0.3	11.8 ± 0.3
**PND-0 Anogenital distance (mm)**				
Males	3.44 ± 0.06	--	--	3.54 ± 0.04
Females	1.47 ± 0.02	--	--	1.49 ± 0.02

Table S6. Summary of results from natural breeding of F_1 males to two untreated females.

Variable	Control	500×	1000×	2000×
**Number of F_1 D-males**				
Bred	10	10	10	10
Mated two females	8	7	9	8
Mated one female	2	3	1	2
Impregnated two females	5	4	7	6
Impregnated one female	5	6	3	4
Sired two live litters	4	4	6	6
Sired one live litter	6	6	4	4
Infertile	0	0	0	0
**Mean ± S.E. per fertile male**				
Dam's corpora lutea	16.7 ± 0.6	18.3 ± 0.6	18.1 ± 0.7	16.9 ± 0.6
Dam's implantation sites	14.2 ± 1.1	13.6 ± 0.6	15.2 ± 0.8	15.2 ± 0.2
Dam's live embryos	13.2 ± 1.4	12.8 ± 0.7	14.7 ± 0.8	14.8 ± 0.3
Dam's resorption sites	1.0 ± 0.5	0.8 ± 0.3	0.5 ± 0.2	0.4 ± 0.2
Preimplantation loss (%)	11.1 ± 4.8	22.6 ± 5.2	11.3 ± 2.9	9.3 ± 2.8
Postimplantation loss (%)	11.0 ± 8.0[a]	6.3 ± 2.9	7.6 ± 4.9	2.6 ± 1.0

[a]Includes one outlier control male; both females mated to this male had high resorption rates.

Table S7. Summary of results from F_1 male in utero insemination of untreated females.

**Variable**	**Control**	**500×**	**1000×**	**2000×**
**Number of F_1 C-males**				
Bred	10	10	10	10
Infertile	1	1	2	3
**Mean ± S.E.**				
Dam's corpora lutea	13.7 ± 0.6	14.9 ± 0.8	14.5 ± 0.6	14.5 ± 0.8
Dam's implantation sites	9.0 ± 1.6	9.0 ± 1.8	8.3 ± 1.8	7.8 ± 2.1
Preimplantation loss (%)	37.0 ± 9.7	40.5 ± 10.5	43.4 ± 11.0	48.6 ± 13.0
SP22 (optical density)	0.424 ± 0.015	0.382 ± 0.015	0.364 ± 0.012	0.394 ± 0.018

Table S8. Mean ± S.E. organ weights of F_1 adults at necropsy (males PND 89-93, females PND 96-104).

**Variable**	**Control**	**500×**	**1000×**	**2000×**
**Males**				
Number examined	24	25	24	24
Terminal body weight (g)	489.8 ± 7.1	490.7 ± 6.6	481.3 ± 7.9	394.5 ± 9.4***
Brain (g)	2.04 ± 0.01	2.05 ± 0.02	2.03 ± 0.02	1.97 ± 0.02**
Pituitary (mg)	11.70 ± 0.23	12.27 ± 0.47	12.61 ± 0.32	10.28 ± 0.55*
Thymus (g)	0.68 ± 0.04	0.58 ± 0.03*	0.61 ± 0.03	0.48 ± 0.03**
Lung (g)	1.68 ± 0.06	1.80 ± 0.06	1.73 ± 0.07	1.69 ± 0.07
Liver (g)	19.98 ± 0.47	20.01 ± 0.39	20.01 ± 0.53	16.30 ± 0.53***
Kidneys (g)	3.61 ± 0.08	3.64 ± 0.06	3.71 ± 0.07	3.19 ± 0.07***
Spleen (g)	1.02 ± 0.07	0.89 ± 0.03	1.07 ± 0.07	0.75 ± 0.04**
Adrenals (mg)	61.01 ± 1.29	63.55 ± 1.64	62.15 ± 2.56	54.31 ± 2.00*
Relative weights[a]				
Brain (g)	0.42 ± 0.01	0.42 ± 0.01	0.42 ± 0.01	0.50 ± 0.01
Pituitary (mg)	2.40 ± 0.06	2.51 ± 0.10	2.64 ± 0.08	2.63 ± 0.16
Thymus (g)	0.14 ± 0.01	0.12 ± 0.01	0.12 ± 0.01	0.12 ± 0.00
Lung (g)	0.34 ± 0.01	0.38 ± 0.02	0.34 ± 0.01	0.42 ± 0.02
Liver (g)	4.08 ± 0.07	4.08 ± 0.07	4.15 ± 0.08	4.13 ± 0.08
Kidneys (g)	0.74 ± 0.01	0.74 ± 0.01	0.77 ± 0.01	0.81 ± 0.01
Spleen (g)	0.21 ± 0.01	0.19 ± 0.01	0.21 ± 0.01	0.19 ± 0.01
Adrenals (mg)	12.48 ± 0.26	12.99 ± 0.35	13.00 ± 0.58	13.84 ± 0.48
**Females**				
Number examined	24	24	24	24
Terminal body weight (g)	346.7 ± 7.1	343.1 ± 5.5	341.5 ± 5.9	287.3 ± 6.4***
Brain (g)	1.85 ± 0.02	1.84 ± 0.02	1.82 ± 0.05	1.78 ± 0.02
Pituitary (mg)	15.82 ± 0.52	14.50 ± 0.59	15.37 ± 0.57	14.20 ± 0.51
Thymus (g)	0.32 ± 0.03	0.33 ± 0.02	0.35 ± 0.03	0.24 ± 0.01
Lung (g)	1.60 ± 0.10	1.46 ± 0.09	1.43 ± 0.09	1.27 ± 0.08
Liver (g)	15.39 ± 0.42	15.71 ± 0.28	15.49 ± 0.42	13.34 ± 0.36***
Kidneys (g)	2.36 ± 0.07	2.37 ± 0.05	2.54 ± 0.07	2.43 ± 0.05
Spleen (g)	0.73 ± 0.03	0.76 ± 0.05	0.78 ± 0.06	0.61 ± 0.05
Adrenals (mg)	84.17 ± 3.18	79.88 ± 2.29	85.35 ± 4.76	70.74 ± 2.52*
Relative weights[a]				
Brain (g)	0.54 ± 0.01	0.54 ± 0.01	0.53 ± 0.01	0.63 ± 0.02
Pituitary (mg)	4.57 ± 0.13	4.24 ± 0.16	4.50 ± 0.14	4.94 ± 0.14
Thymus (g)	0.09 ± 0.01	0.10 ± 0.01	0.10 ± 0.01	0.08 ± 0.01
Lung (g)	0.45 ± 0.03	0.43 ± 0.02	0.42 ± 0.02	0.45 ± 0.04
Liver (g)	4.43 ± 0.07	4.59 ± 0.08	4.53 ± 0.08	4.64 ± 0.06
Kidneys (g)	0.68 ± 0.02	0.69 ± 0.01	0.74 ± 0.02**	0.85 ± 0.01***
Spleen (g)	0.21 ± 0.01	0.22 ± 0.01	0.23 ± 0.02	0.21 ± 0.01
Adrenals (mg)	24.35 ± 0.88	23.32 ± 0.63	25.15 ± 1.44	24.59 ± 0.60

*Significantly different from control value (p<0.05).

**Significantly different from control value (p<0.01).

***Significantly different from control value (p<0.001).

[a]Relative organ weight per 100 g body weight.

Table S9. Incidences of nephropathy, adrenal cortical hypertrophy (in the zona glomerulosa), and atrophy (in the zona reticularis) in P_0 females at 26 days postpartum.

**Variable**	**Control**	**500×**	**1000×**	**2000×**
Number of P_0 females examined	25	25	25	24
Adrenals				
Hypertrophy, zona glomerulosa	4 (16%)	7 (28%)	7 (28%)	11 (46%)*
Atrophy, zona reticularis	3 (12%)	9 (36%)	10 (40%)	23 (96%)***
Kidney				
Nephropathy	7 (28%)	5 (20%)	11 (46%)	17 (71%)**

*Significantly different from control value ($p<0.05$).

**Significantly different from control value ($p<0.01$).

***Significantly different from control value ($p<0.001$).

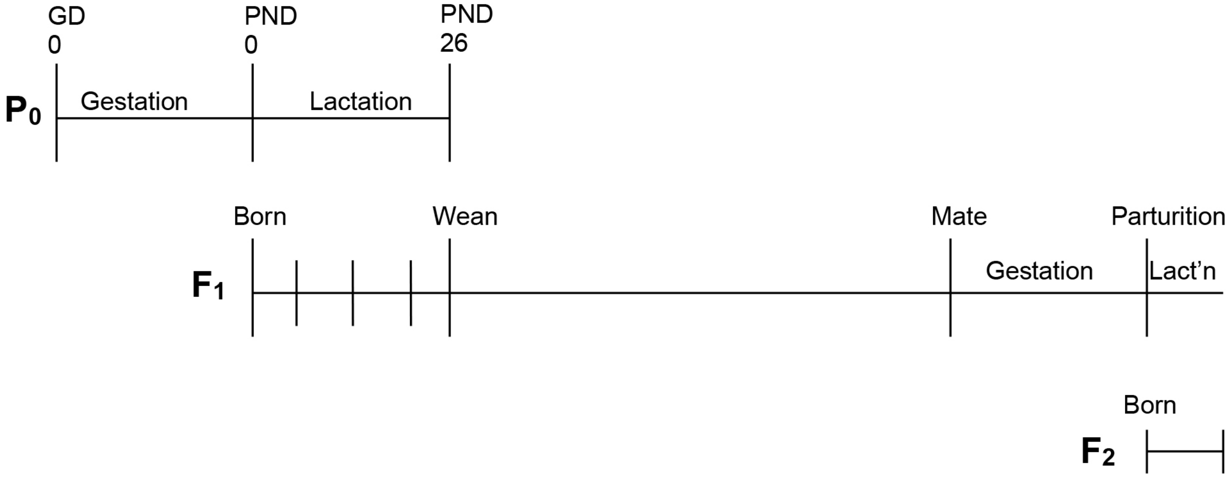

Figure S1. General time-line of the multigenerational bioassay. Parental females (P_0 generation) were obtained timed-pregnant on gestation day (GD) 0. Treatment began on GD 0 and continued to the remainder of the experiment. The P_0 dams delivered their litters (F_1 generation) which were examined at postnatal day (PND) 0 (birth), 6, 13, 21, and 26. Litters were weaned at PND 26 and select F_1 offspring were maintained to adulthood and bred to produce the F_2 generation. F_2 litters were examined at PND 0 and 6.
